# Cerebellum, Predictions and Errors

**DOI:** 10.3389/fncel.2018.00524

**Published:** 2019-01-15

**Authors:** Laurentiu S. Popa, Timothy J. Ebner

**Affiliations:** Department of Neuroscience, University of Minnesota, Minneapolis, MN, United States

**Keywords:** Purkinje cell, simple spike, complex spike, kinematics, performance error, sensory prediction error, forward internal model, generative model

## Abstract

Making predictions and validating the predictions against actual sensory information is thought to be one of the most fundamental functions of the nervous system. A growing body of evidence shows that the neural mechanisms controlling behavior, both in motor and non-motor domains, rely on prediction errors, the discrepancy between predicted and actual information. The cerebellum has been viewed as a key component of the motor system providing predictions about upcoming movements and receiving feedback about motor errors. Consequentially, studies of cerebellar function have focused on the motor domain with less consideration for the wider context in which movements are generated. However, motor learning experiments show that cognition makes important contributions to motor adaptation that involves the cerebellum. One of the more successful theoretical frameworks for understanding motor control and cerebellar function is the forward internal model which states that the cerebellum predicts the sensory consequences of the motor commands and is involved in computing sensory prediction errors by comparing the predictions to the sensory feedback. The forward internal model was applied and tested mainly for effector movements, raising the question whether cerebellar encoding of behavior reflects task performance measures associated with cognitive involvement. Electrophysiological studies based on pseudo-random tracking in monkeys show that the discharge of Purkinje cell, the sole output neurons of the cerebellar cortex, encodes predictive and feedback signals not only of the effector kinematics but also of task performance. The implications are that the cerebellum implements both effector and task performance forward models and the latter are consistent with the cognitive contributions observed during motor learning. The implications of these findings include insights into recent psychophysical observations on moving with reduced feedback and motor learning. The findings also support the cerebellum’s place in hierarchical generative models that work in concert to refine predictions about behavior and the world. Therefore, cerebellar representations bridge motor and non-motor domains and provide a better understanding of cerebellar function within the functional architecture of the brain.

## Introduction

Yogi Berra and Niels Bohr agreed: “Predictions are very hard, especially when they are about the future” (Stanislaw, 1976; Wilford, 1991). It turns out they could be ubiquitous throughout the brain too.

**Figure 1:**
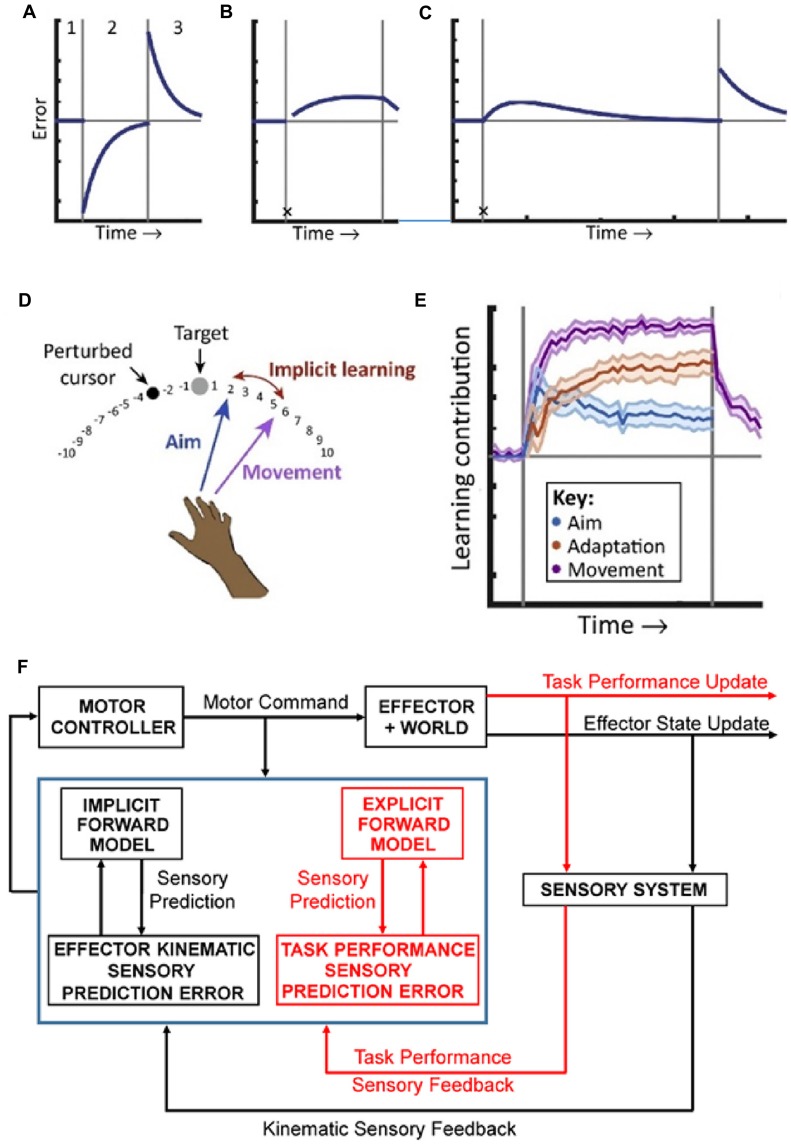
Implicit and explicit mechanisms of motor adaptation. **(A)** The canonical motor-learning curve, including baseline (period 1), adaptation to a sensorimotor perturbation (period 2), and return to baseline (period 3). **(B)** Following the first trial after introducing the perturbation (denoted by the black X), subjects are taught to compensate for the rotation by aiming away from the target, towards an additional marker, resulting in immediate task success. In subsequent trials, performance deteriorates due to implicit learning. **(C)** In an extended training period, task performance is eventually restored by strategy adjustments. An after-effect, indicative of implicit learning is revealed when the participants aim directly to the target and the perturbation is turned off. **(D)** Measuring strategy use during adaptation to a visuomotor rotation task. Before movement, participants explicitly report their aim. The implicit learning magnitude is the difference between aiming angle and actual end-point angle. **(E)** The explicit strategy (Aim) is responsible for a large immediate contribution following the perturbation that declines with time. Implicit learning (Adaptation) is slower and monotonic and matches the magnitude of the initial aftereffect. Adapted with permission from McDougle et al. (2016). **(F)** Schematics of the forward internal model hypothesis. Based on inputs from the motor cortex (Motor Command) and sensory system (Sensory Feedback), the cerebellar cortex (symbolized by the blue box) implements two independent forward models, an implicit one for the effector (coded in black) and an explicit one for the task strategy (coded in red). These models provide sensory predictions in two different spaces: one effector-related (kinematic predictions) and one task-related (task performance predictions). These sensory predictions are compared with the correspondent sensory feedback to compute sensory prediction errors in both spaces. Sensory prediction errors are used to independently update each internal model. The cerebellar output, integrating all sensory prediction errors, is used to update the Motor Controller.

It has been hypothesized that central to brain function is learning to make predictions about behavior and the world. The use of predictions to control behavior relies on computing prediction errors, the differences between predictions and reality. Early on, motor control research strongly embraced the importance of generating predictions about upcoming movements using the framework of forward internal models of effectors. Recent work has emphasized that controlling and learning self-directed motor behaviors involves both an implicit forward model of the effector and an explicit model of the task (Taylor and Ivry, 2011; Streng et al., 2018b; see Figure 1F). Multiple forward models require a more nuanced view on the error sources. Further, self-directed motor behavior cannot be cleanly separated from the underlying cognitive context.

The cerebellum is an integral part of the motor control system and is thought to be geared to predicting aspects of upcoming motor behavior and involved in processing prediction errors. This review focuses on the cerebellum’s role in implementing forward internal models and examines whether the discharge of cerebellar neurons have the requisite predictive and feedback signals essential for generating prediction errors. Importantly, the review examines whether the signals encoded are restricted to information only about effectors or whether the signals include task-related information.

## Predictive Prowess of the Motor System and Forward Internal Models

Motor behavior, being amenable to precise measurement and manipulation of well-defined parameters of movement, showcases the nervous system’s ability to anticipate motor outcomes over a wide range of behaviors and experimental conditions. For example, during a saccade there is neither visual nor proprioceptive sensory feedback (Keller and Robinson, 1971; Guthrie et al., 1983; Thiele et al., 2002). Yet, the variability in the motor command, as reflected in eye movement velocity, is corrected to maintain saccade accuracy (Golla et al., 2008; Xu-Wilson et al., 2009). Similarly, the neural machinery generating saccades compensates for perturbations due to blinking (Rottach et al., 1998). Therefore, in the absence of sensory feedback, the control of saccadic eye movements relies on predicting the consequences of motor commands rather than sensory feedback. The anticipatory grip forces on an object when predictable loads are applied to the arm (Johansson and Cole, 1992; Flanagan and Wing, 1997) are also consistent with making predictions about arm movements and the associated inertial forces (Kawato, 1999). Adaptation to perturbations, such as force fields or visuomotor transformations, provides compelling evidence that the brain learns to anticipate the consequences of motor commands (Shadmehr and Mussa-Ivaldi, 1994; Thoroughman and Shadmehr, 1999). Similarly, a persuasive framework for hand-eye coordination requires the anticipation of effector kinematics (Scarchilli et al., 1999). In addition, the CNS predicts the effects of common environmental constraints, for example gravitation (Zago et al., 2004; Lacquaniti et al., 2013).

The ubiquitous predictions observed during motor psychophysical experiments have to be integrated into a wider range of control processes including compensating for the inherent delays in sensory feedback, countering sensory reafferent signals, state estimation, and motor learning. Internal models offer a widely accepted computational framework for these control requirements by providing neural representations of the input-output relationships or their inverses for specific elements of the motor plant or properties of the environment to be controlled (Kawato, 1999). With the motor command and current sensory information as its inputs, a forward internal model predicts the consequences of motor actions (Jordan and Rumelhart, 1992; Miall et al., 1993; Miall and Wolpert, 1996). Forward model predictions can be compared to the actual sensory feedback to compute the difference between the intended and achieved action. This difference is termed a sensory prediction error. In turn, sensory prediction errors are used to control movements online, cancel sensory reafference due to self-generated movement, perform state estimation, guide motor learning, and update the forward model (Jordan and Rumelhart, 1992; Miall et al., 1993; Wolpert et al., 1995; Doya, 1999; Shadmehr et al., 2010). In support of this concept, sensory prediction errors have been shown to be used in motor adaptation across different effectors and behaviors (Wallman and Fuchs, 1998; Noto and Robinson, 2001; Morton and Bastian, 2006; Tseng et al., 2007; Xu-Wilson et al., 2009).

## Cerebellum as a Forward Internal Model

Many investigators have hypothesized that internal models of the motor system, in general, and forward models, specifically, are acquired and maintained in the cerebellum (Miall et al., 1993; Shidara et al., 1993; Shadmehr and Holcomb, 1997; Wolpert et al., 1998; Kawato, 1999; Imamizu et al., 2000; Pasalar et al., 2006; Taylor et al., 2010; Popa et al., 2013). As a large body of literature supports this hypothesis, we summarize only a few key findings. The motor deficits in patients with cerebellar disorders are consistent with corrupted forward models, including loss of saccade accuracy due to motor command variability (Golla et al., 2008; Xu-Wilson et al., 2009), inability to adapt reaching movements to motor perturbations such as force fields or visuomotor rotations (Maschke et al., 2004; Tseng et al., 2007; Taylor et al., 2010) and selective disruptions of predictive adjustments during split belt locomotion (Bastian, 2006; Morton and Bastian, 2006).

In healthy subjects, functional imaging reveals changes in cerebellar activation following motor learning, further supporting the postulate that the cerebellum is the locus for the acquisition and storage of internal models of the musculoskeletal system (Shadmehr and Holcomb, 1997; Imamizu et al., 2000; Diedrichsen et al., 2005; Bursztyn et al., 2006; Tseng et al., 2007). To illustrate with a specific study, one experiment required participants to perform a ballistic hand movement and use their thumb to press a button at a fixed time interval relative to movement onset. The results reveal that control of the thumb was based on an internal representation of relative time if the time interval was longer than the movement period. Conversely, thumb control was based on a state estimation of the arm if the time interval overlapped with the arm movement. Consistent with the forward model hypothesis, the cerebellum was selectively activated when state estimation was required by the task (Diedrichsen et al., 2007). Imaging studies also demonstrate strong cerebellar activation by motor errors, both task performance errors (Flament et al., 1996; Imamizu et al., 2000; Diedrichsen et al., 2005; Grafton et al., 2008) and sensory prediction errors (Schlerf et al., 2012). These are essential signals for the formation and modification of internal models. Finally, transient cerebellar disruption using transcranial magnetic stimulation induces movement perturbations that can be accounted for by the cerebellum making a prediction of the kinematic state of the arm at a lead time of 130 ms (Miall et al., 2007), as expected if the cerebellum implements a forward internal model.

## Implicit and Explicit Contributions to Motor Learning

Explicit or declarative information processing occurs under conscious control, for example following a verbal instruction on how to execute a motor task. Implicit or procedural information processing is automatic and manifest in skill performance, such as experience driven improvement in motor output (Haith and Krakauer, 2018). Historically, motor learning was considered as purely an implicit process, solely based on updating an effector forward internal model (Figure 1A). However, during motor adaptation, there are both explicit and implicit contributions with different effects and implications for cerebellar forward internal models.

An elegant experiment successfully decoupled the contributions of implicit and explicit processes on motor learning. In a reaching task the visual feedback was perturbed by introducing a constant angular rotation between the hand and cursor positions. In the second trial after introducing this visuomotor rotation, the subjects were instructed to change the aim of the movement to compensate for the visual perturbation. This explicit strategy immediately restored task performance by minimizing the end-point errors, defined as the angular distance between target and cursor. However, in subsequent trials end-point errors gradually increased, reflecting the normal motor adaptation that occurs during visuomotor rotation (Mazzoni and Krakauer, 2006; Figure 1B).

This unexpected result challenged the canonical view of motor learning and required a closer look at motor errors. Implicit sensory prediction errors, defined as the difference between the forward model predictions of the kinematics of the arm and the corresponding sensory feedback and, measured in this experiment as the difference between direction of arm movement and direction of cursor movement, are maximal in early perturbed trials. In contrast, end-point error is minimal because the explicit strategy counteracts the perturbation. The findings demonstrate that sensory prediction errors computed in effector-related space drive implicit motor adaptation, as predicted by the forward internal model hypothesis and that implicit learning occurs relatively independently from task performance, as defined by the end-point errors. Conversely, a task performance measure is computed in a task-related space and reflects both the kinematic sensory prediction errors and the explicit strategy. The results establish a functional segregation between an effector-related domain involved in implicit processes and a task-related domain related to explicit strategies. However, when adaptation is allowed to progress over a large number of trials, task performance errors plateau and then decrease (Taylor and Ivry, 2011; Figure 1C). This non-monotonic distribution of task performance allows for several possible explanations. One would be that subjects decide to disregard the instructions received. This would result in abrupt changes in task performance unlike the gradual recovery of the end-point errors observed. A model that fits the data adds to the implicit learning driven by kinematic error prediction, an explicit learning strategy driven by task performance. In this scenario, the implicit and explicit adaptation processes compensate each other to restore task accuracy.

To further elucidate the contribution of explicit strategies, in a series of newer studies of adaptation to visuomotor rotation, subjects were required to report their reach aim before movement in the absence of prior knowledge about the perturbation (Taylor et al., 2014; Bond and Taylor, 2015). In contrast with previous studies (Taylor and Ivry, 2011), the declarative contribution was measured by the verbally reported aim direction. The difference between aim and end point directions served as a measure of implicit adaptation, as the subjects endeavored to reach the target (Figure 1D). In these conditions an explicit strategy emerges, counteracting the imposed rotation and contributing in parallel with the implicit adaptation to the motor learning, consistent with the observations of the previous experiment. The implicit adaptation is a slow monotonic process and context independent as it is driven by sensory prediction errors computed in an effector centered domain. The explicit learning is faster, driven by task performance errors, exploratory, responsive to changing task demands, accounts for a large fraction of the improvement throughout the learning process (Figure 1E) and involves the cerebral cortex as diminished prefrontal function increases task error drift (Taylor and Ivry, 2014; Taylor et al., 2014; Bond and Taylor, 2015). The implicit process is thought to reflect the updating of a cerebellar forward model of the arm while the explicit strategy is thought to be under frontal control, at least in the early phase of learning, and reflects strategic planning and action selection (McDougle et al., 2016). Patients with cerebellar pathology when given a declarative strategy that accounts for the visuomotor rotation lack the after effects associated with motor adaptation and lack the gradual degrading in task performance present in the healthy subjects, confirming that intact cerebellar function is required for implicit motor adaptation (Taylor et al., 2010). One surprising observation is that the emergence of the explicit strategy depends on the cerebellar function (Butcher et al., 2017). As motor adaptation unfolds in both implicit and explicit domains, this raises the question whether are there representations of both of these processes in the discharge of cerebellar neurons?

## Information Processing in Cerebellar Neurons for Implicit and Explicit Forward Internal Models

Strong support for cerebellar involvement in forward models and in computing sensory prediction errors is emerging from studying the activity of cerebellar neurons. The output of a well-tuned forward model should be relatively insensitive to sensory reafferents due to self-generated movements, as the predictions should closely match the sensory feedback. Conversely, the output should be highly sensitive to passive movements or unexpected perturbations. During passive movement sensory feedback dominates in the absence of motor commands and during perturbations the model predictions predicted will be poorly matched with the actual feedback. These expectations for a forward internal model were successfully tested in the rostral fastigial nucleus. The discharge of these neurons have higher sensitivity to passive movements compared to comparable self-generated movements (Brooks and Cullen, 2013). Moreover, when the head movement is perturbed by external forces, the initially high sensitivity of fastigial neurons gradually decreases, mirroring the adaptation that occurs in head movement (Brooks et al., 2015). Together these results are consistent with the output of an effector forward model that adapts its predictions to minimize sensory prediction errors.

What about the discharge of Purkinje neurons, the final stage in the information processing of the cerebellar cortex and the only output? Within the framework of the forward internal model hypothesis, we proposed that Purkinje cell firing represents both the predictions of the motor command consequences and the corresponding sensory feedback (Popa et al., 2013, 2016a). For an effector forward model, both the prediction and the sensory feedback are thought to be expressed in the effector kinematics space (Wolpert et al., 1995; Miall and Wolpert, 1996).

Purkinje cell simple spike (SS) discharge modulates with and is correlated to upcoming eye and arm kinematics in a variety of motor behaviors (for reviews, see Ebner and Pasalar, 2008; Ebner et al., 2011). Conversely, SSs modulate with limb kinematics during passive movements, arguing for sensory feedback encoding. The wide timing distribution of SS firing relative to movement, spanning both feedforward and feedback timing with a mean hovering around 100 ms prior to movement, suggests a bias in favor of predictive kinematic representations (Hewitt et al., 2011). More compelling evidence for a forward model is the observation that SS activity is strongly linked to the kinematic consequences of the motor commands and not to the dynamic output of the motor plant (Pasalar et al., 2006). Also, kinematic representations in the SS firing are conserved across different behaviors (Roitman et al., 2005; Hewitt et al., 2011). These findings offer support, but not proof, of the concept that the cerebellar cortex realizes a forward model of the arm.

A better understanding of the nature and temporal aspects of Purkinje cell representations requires a task that imposes robust and sustained online error processing and allows a decoupling of past and future states. These requirements were fulfilled by using a pseudo-random, manual tracking task (Hewitt et al., 2011; Popa et al., 2012, 2017). Purkinje cell recordings during pseudo-random tracking confirm that SS firing encodes arm movement kinematics including position, velocity, and acceleration (Hewitt et al., 2011; Popa et al., 2012, 2017; Streng et al., 2017). The use of linear regression analyses in which we first removed the contribution of all motor parameters from the SS firing except the parameter of interest and then evaluated the relation between the parameter of interest and the residual SS firing show that these kinematic signals are independently represented, and that individual Purkinje cells simultaneously encode several kinematic parameters (Popa et al., 2012, 2017).

**Figure 2:**
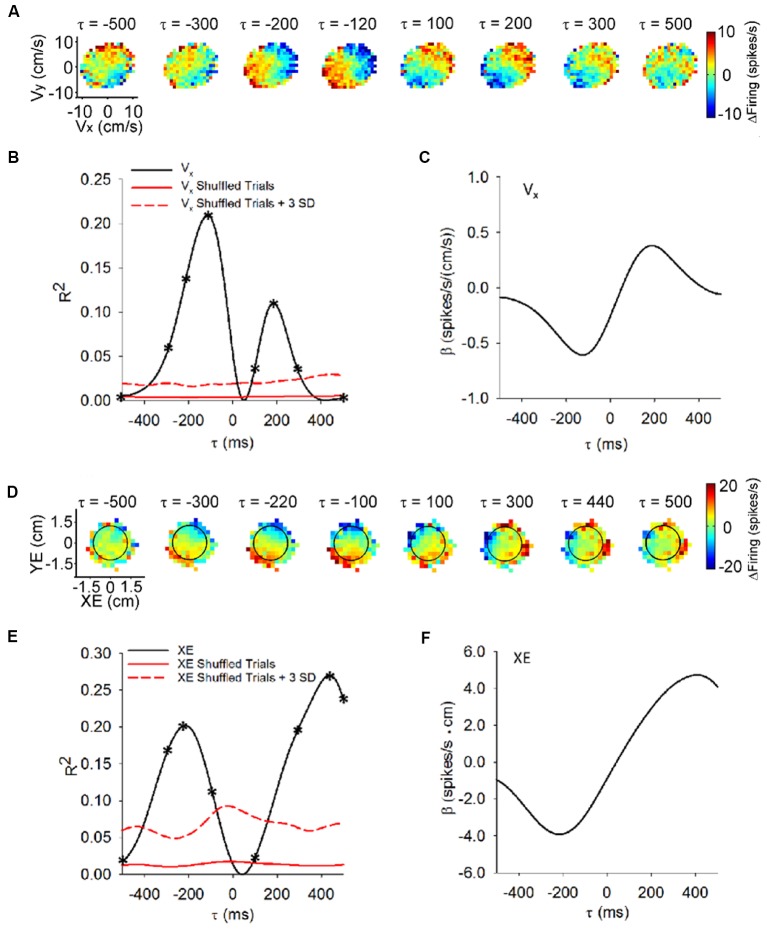
Time course of the simple spike (SS) modulation with behavioral parameters during pseudo-random tracking. **(A)** Color coded maps of the SS firing, relative to the overall mean, for an example Purkinje cell in the velocity space (V_x_, V_y_) at different lead/lags (τ). Negative τ represents the firing leading velocity. **(B)** For the cell in **(A)**, the R^2^ for V_x_ as a function of lead/lag (τ) reveals modulation at both feedforward and feedback timing. The red trace shows the mean of the control regressions computed on trial shuffled data (100 repetitions). The dashed red trace is the mean +3 SD of the control regressions. On the R^2^ temporal profiles asterisks (*) indicate the leads/lags of the corresponding SS firing maps in **(A)**. **(C)** For the same neuron, the regression coefficients for V_x_ (${\text{β}}_{{\text{V}}_{\text{X}}}$) are plotted as a function of τ. The sign change in ${\text{β}}_{{\text{V}}_{\text{X}}}$ represents the reversal in the firing sensitivity at feedforward lead compared to feedback lag. **(D)** SS firing maps of another example Purkinje cell with position error (XE, YE) at different leads/lags (τ). Target depicted by black circles. Same conventions as in **(A)**. **(E,F)** For the cell in **(D)**, the temporal profiles for the R^2^ **(E)** and regression coefficients (β_XE_) for XE **(F)** exhibit predictive and feedback local maxima. β_XE_ shows the reversal in the SS firing sensitivity at lead compared to lag timings **(F)**. Conventions for red lines as in **(B)**. Adapted with permission from Popa et al. (2016a).

The utility of the pseudo-random tracking paradigm is best revealed by establishing the predictive and feedback encoding of kinematics by Purkinje cells (Popa et al., 2012, 2017). The predictive and feedback modulation is illustrated in the sequence of firing maps of SS modulation with velocity across a range of time shifts (i.e., τ-values) as shown in Figure 2A. In this example, SS firing relative to the mean firing precedes hand velocity, with higher firing in the lower left quadrant that reaches a maximum at a feedforward timing of −120 ms. At feedback lag, a reciprocal SS modulation pattern emerges, with peak firing in the upper right quadrant at approximately 200 ms. Temporal linear regressions of the SS discharge with each behavioral parameter provide quantitative measures of the temporal relationship (τ-value) and the correlation strength (R^2^ and regression coefficient-β; Hewitt et al., 2011; Popa et al., 2012). For this Purkinje cell, the velocity R^2^ and β profiles (Figures 2B,C, respectively) characterize the feedforward and feedback SS encoding, with local maxima at the leads and lags corresponding to the timing of the maximal modulations in the firing maps (Figure 2A). The lead and lag correlations are well above chance, as determined by regressions of the trial randomized data. Approximately, 70% of Purkinje cells exhibit this bi-modal profile with kinematics. We interpret these SS modulation profiles as the predictive and feedback constituents of the sensory prediction error computed by an implicit forward internal model of the arm.

Importantly, pseudo-random tracking provides additional insights into Purkinje cell representations. As the monkeys track the moving target, they attempt to maintain the cursor in the target center. The task also requires that the monkeys correct cursor excursions outside the target within 500 ms. This provides for several natural and continuous measures of performance errors including position error (components of the position error vector defined by the cursor and target center positions), radial error (magnitude of the position error vector) and direction error (angle between the current cursor position and the target center; Popa et al., 2012, 2017). The SS firing encodes these error parameters using bimodal, predictive-feedback representations. The firing maps of a Purkinje cell (Figure 2D) shows that the SS discharge leads position error from −300 ms to −100 ms, as the highest firing occurs in the lower left quadrant. The SS firing also lags position error from 300 ms to 500 ms when the highest firing occurs in the upper right quadrant. The R^2^ and β profiles (Figures 2E,F, respectively) for the x-component (XE) of position error have local maxima at −220 and 440 ms (Figures 2E,F; respectively). For a large majority of the Purkinje cells, the SS discharge signals at least one of these performance error parameters.

At the population level, the strength of performance error encoding is robust and comparable to the encoding of kinematics. Also, there is no segregation of Purkinje cells into error or kinematic subpopulations, showing that the integration of the task errors and kinematics occurs at the individual cell level (Popa et al., 2012). As mentioned before, the pseudo-random tracking task uncouples the past and future behavioral states, thus unveiling that the SS dual representations of individual error parameters, including a pair of predictive and feedback signals with opposing modulations, are ubiquitous in the Purkinje cells population (Popa et al., 2012). As these performance error signals are task-related and independent of kinematics, we interpret these modulation profiles as the feedforward and feedback elements computed by a forward internal model of a task specific, explicit strategy (Popa et al., 2013, 2014).

**Figure 3:**
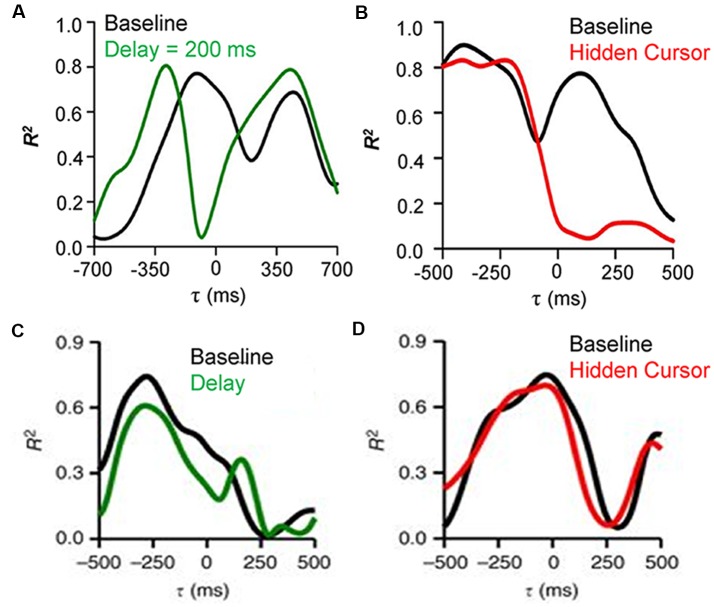
Effects of feedback manipulations on behavioral parameters encoding by SS discharge. **(A)** R^2^ temporal profiles for an example Purkinje cell SS firing regressed with position error during delay cursor delay (baseline—black trace, 200 ms delay—green trace). The predictive encoding shifts to more negative τ-values while the timing of the feedback modulation does not change. **(B)** R^2^ temporal profiles for an example Purkinje cell SS firing regressed with position error during the hidden cursor condition (baseline—black trace, hidden cursor—red trace). The reduction in visual feedback decreases the strength of the feedback encoding of position error but not the predictive encoding. **(C)** R^2^ temporal profiles for an example Purkinje cell SS firing regressed with velocity during cursor delay (baseline—black trace, 100 ms delay—green trace). **(D)** R^2^ temporal profiles for an example Purkinje cell SS firing regressed with velocity during the hidden cursor condition (baseline—black trace, hidden cursor—red trace). The kinematic representations are not changed by either manipulation of the visual feedback. Adapted with permission from Streng et al. (2018b).

To establish that Purkinje cells provide the output of a forward internal model of performance errors it needs to be shown that the feedforward signals are the predicted consequences of the motor commands while the feedback signals reflect the sensory input. To test these requirements, Purkinje cell recordings during a modified pseudo-random tracking involving two perturbations of the visual feedback (Streng et al., 2018b). The first manipulation introduced delays between hand and cursor movements. If the feedforward modulation of position error is driven by the motor commands, the prediction will occur earlier relative to the cursor and the temporal shift should match the imposed delay. However, the feedback signal timing, being based on visual sensory input, will not be affected. The results confirmed these expectations, as shown for an example Purkinje cell in which the cursor delay shifts the predictive timing of the position error modulation, as determined by the local maxima in the R^2^ temporal profile, to more negative τ-values while the timing of the feedback modulation does not change (Figure 3A). The second manipulation hid the cursor while inside the target, thus reducing the visual feedback during task execution (Hidden cursor condition). The expectations were that the SS feedback modulation inside the target will be reduced as a result of reduced visual input, while the predictive modulation, driven by efferent copies of the motor command will not be affected. Again, the experimental findings confirmed the expectations based on a forward internal model of performance errors, with a decrease in the strength of the feedback encoding of position error but not the predictive encoding (Figure 3B). Moreover, the kinematic representations were not affected by either manipulation (Figures 3C,D), confirming the independence of the error and kinematic representations in the SS firing.

**Figure 4:**
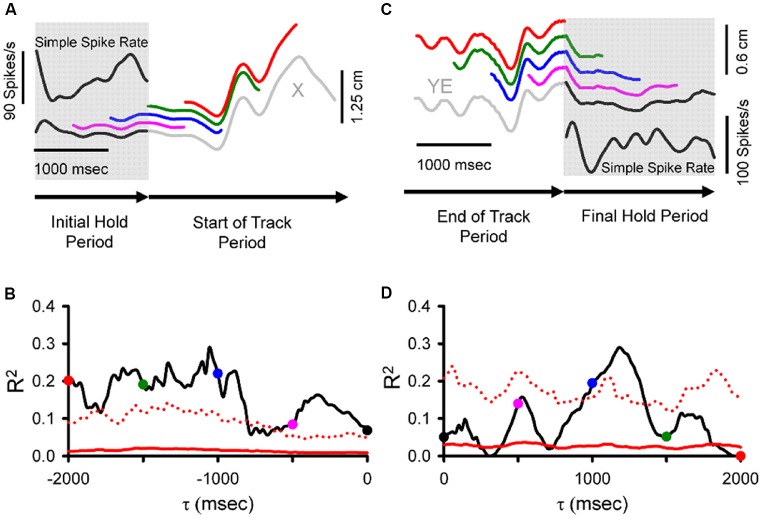
During hold periods SS firing correlates with movement parameters during track period. **(A)** SS firing (inset) during the initial hold period prior to tracking (gray shadow) from an example trial matched to position (specifically X-position) at τ values spanning 0 to −2,000 ms illustrated by the sliding window. Note that the window length is equal in duration to the initial hold period. Colored traces illustrate the X sliding window at different times: black (0 ms), pink (−500 ms), blue (−1,000 ms), green (−1,500 ms), and red (−2,000 ms). **(B)** For the SS discharge of this neuron, the R^2^ obtained from correlating firing rate with X-position across all trials is shown as a function of time (τ). The key observation is that the SS firing in the hold period encodes information about the upcoming position. **(C)** SS discharge rate (inset) during the final hold period (gray shadow) matched to position error (specifically YE) recorded in both track (gray) and final hold (black) periods using a sliding window of the same duration as the final hold period spanning from 0 to 2,000 ms. Colored traces illustrate the YE sliding window at different times: black (0 ms), pink (500 ms), blue (1,000 ms), green (1,500 ms), and red (2,000 ms). **(D)** For this Purkinje cell, plot of the R^2^ as a function of time (τ) from regressing SS with YE across all trials. Here the critical observation is that the firing in the hold period contains position error information about the just completed track period. Direction of recording time is indicated by bottom arrows in **(A,C)**. For **(B,D)**, conventions for the colored dots conventions are as in **(A,C)**, respectively. Chance encoding (red traces) and conventions for τ values, as in Figure 2. Adapted with permission from Popa et al. (2017).

The results described above focused on the SS activity centered on current movement (±500 ms). However, the pseudo-random tracking task also allowed a determination of the relation between Purkinje cell firing and the behavior over longer time intervals (Popa et al., 2017). Both kinematic and task performance parameters were found to be represented in the SS firing at leads and lags spanning 2,000 ms before to 2,000 ms after the movement. We refer to these extended predictive and feedback modulations as “long-range signals.” During tracking, these long-range signals allowed decoding of the individual behavioral parameters, kinematic and task performance, with remarkable accuracy, well above the random level. Moreover, during the periods preceding (Figures 4A,B) and following tracking (Figures 4C,D), when the monkeys were required to hold the cursor within a stationary target, the SS activity encoded expectations or memories of both kinematics and performance errors (Popa et al., 2017).

The long-range signals of effector states and task performance following a movement are consistent with a form of working memory that can bridge the inter-trial intervals as expected by the cerebellar involvement in generating the explicit strategy. The long-range predictive signals are consistent with planning and action expectations that could be used to seed the forward model acquisition. Long-range preparatory Purkinje cells activity could be used for evidence accumulation prior to movement, as observed in a task requiring mice to make a left-right decision (Deverett et al., 2018). Support for these long-range signals likely involves the cerebellum’s closed-loops connections with numerous regions in the cerebral cortex (for reviews, see Schmahmann and Pandya, 1997; Strick et al., 2009; Bostan et al., 2013; Lena, 2016). Further, a network relying on hippocampal-cerebellar interactions is involved in learning sequence-based navigation in mice (Babayan et al., 2017). These findings argue for the cerebellar involvement in persistent activity loops with the cerebral cortex related to higher functions.

## Role for Complex Spikes in Predictions, Error Processing and Forward Models

One of the more prominent hypotheses of cerebellar function is that complex spikes (CSs) are the sole conduits of error information and those error signals drive motor learning (Marr, 1969; Albus, 1971; Oscarsson, 1980; Ito and Kano, 1982). While supported by several studies (for reviews, see Ito, 2002; Gao et al., 2012), the hypothesis that CS discharge primarily provides error information critical for motor learning does not cover accurately the spectrum of experimental observations. Many studies failed to find error signals in the firing of inferior olivary neurons or in the CS discharge (for reviews, see Catz et al., 2005; Llinás, 2014; Popa et al., 2016b; Streng et al., 2018a). In addition there are multiple demonstrations of cerebellar learning that is independent of climbing fiber input (Boyden et al., 2004; Ke et al., 2009; Nguyen-Vu et al., 2013; Shin et al., 2014; Hewitt et al., 2015).

CSs also convey parametric information related to movements contrasting with the error signaling hypothesis. For example, climbing fiber input modulates with eye and head movements induced by vestibulo-ocular rotation in the dark, when the retinal slip is absent (Winkelman et al., 2014), with movement kinematics during ocular pursuit (Kobayashi et al., 1998) and with reach kinematics (Fu et al., 1997; Kitazawa et al., 1998). Moreover, during pseudo-random tracking CS firing modulates strongly with arm kinematics including position, velocity, and acceleration, showing that climbing fiber input signals movement information beyond motor errors (Streng et al., 2017).

Although typically thought to be primarily driven by feedback errors, during pseudo-random tracking, climbing fiber modulation leads both kinematics and performance errors (Streng et al., 2017). Furthermore, at the population level feedforward CS activity is more frequent than feedback modulation. Others have observed this predictive property, as CSs modulate with eye performance inferred errors (Frens et al., 2001; Winkelman and Frens, 2006; Winkelman et al., 2014), anticipatory errors in eye blink conditioning (Ohmae and Medina, 2015) and with learned sensorimotor predictions of reward (Heffley et al., 2018). Together these observations demonstrate the need to reconsider the view that CSs only convey information about feedback errors and acknowledge the robust kinematic information carried by the climbing fiber input. Therefore, the dominant view that SS and CS discharge carry functionally unique signals cannot withstand a detailed examination.

The roles played by climbing fiber input in motor learning and error signaling are under reconsideration (Catz et al., 2005; Streng et al., 2018a). Taking into account that spontaneous CS firing is essential for cerebellar function and climbing fiber input results in a global depolarization that is likely to alter how Purkinje cells process parallel fiber input (for review, see Kitamura and Kano, 2013; Streng et al., 2018a), we hypothesized that climbing fiber input to Purkinje cells modulates the information present in the SS firing (Streng et al., 2017). An examination of the SS firing encoding uncovered that CSs trigger robust, step-like changes in the kinematic and position error signals present in the SS discharge. This control over a Purkinje cell’s encoding state is hypothesized to optimize motor performance and/or compensate for drifts in the SS representations and is consistent with climbing fiber input providing both error and non-error information as well as predictive encoding (Streng et al., 2018a). These findings also account for spontaneous CSs firing as a mechanism to provide SS encoding homeostasis. Consistent with the CSs playing a homeostatic role in spontaneous SS firing, earlier studies showed that removal or stimulation of climbing fiber input produces dramatic and long-term changes in the SS firing (Colin et al., 1980; Montarolo et al., 1982; Cerminara and Rawson, 2004). Moreover, the rapid changes in SS encoding suggest that CS discharge directs an internal model selection process, allowing cerebellar cortical output to accommodate to changes in behavioral conditions.

## Implications for Cerebellum Providing Both Implicit and Explicit Models

The encoding of kinematic and performance errors in the discharge of Purkinje cells supports the simultaneous presence of cerebellar effector and task-specific forward models. When visual feedback was disrupted, the predictive and feedback SS modulations are mismatched (Streng et al., 2018b). However, the animals can still perform the task and previous psychophysical studies found that the motor system continues to generate accurate predictions during altered visual feedback (Kumar and Mutha, 2016). The invariance in the SS kinematic signals as well as the constancy of the task error predictions (Figure 3) argue that the internal models are making precise estimates of the consequences of the motor command based on the present states of the effector and target, allowing the animals to perform skilled behaviors even with sub-optimal visual feedback.

The independence of arm kinematics and task performance forward models is consistent with recent psychophysical results. When subjects were asked to intercept their moving index finger with the index of the other hand in the absence of visual feedback, there was no difference in performance whether the target finger was moving voluntarily or passively (Darling et al., 2018). This result was interpreted as evidence that forward internal models are not necessary for state estimation. However, the task in this psychophysical study is similar to the hidden condition during pseudo-random tracking by primarily providing sensory feedback about target kinematics. Under these conditions, the effector and task performance forward internal models work in concert to preserve the task performance in noisy conditions.

The presence of arm and task performance forward models integrated at the Purkinje cell level could provide insights into motor learning as presented in Figure 1. The effector forward model operates in the kinematics domain and is consistent with the classical view of implicit motor learning (Figure 1A). The task model operates in the task performance domain and is consistent with a forward model of the explicit strategy (see Figure 1E). In this view, in the initial phase of adaptation only the effector model is updated to exclusively minimize the sensory prediction errors related to kinematic parameters, while the explicit strategy, under cerebral cortical control (McDougle et al., 2016), is conserved. As a result, the implicit learning progresses to the detriment of task performance (Figure 1B). In the late stages of adaptation, the cerebellum acquires and updates forward models of the explicit strategy. Based on the motor command, both forward models provide predictions and compute sensory prediction errors, simultaneously optimizing the effector response and the action outcome (see Figure 1C). This two stage hypothesis for the explicit strategy is also consistent with the observation that working memory load interferes with motor learning in the early phase but not in latter phase of motor learning (Keisler and Shadmehr, 2010).

An important aspect of brain function is skilled performance. Skilled behavior requires fast execution, decreased sensitivity to perturbations and reduced cognitive effort (Ramnani, 2014; Haith and Krakauer, 2018). Skilled behavior is thought to involve the acquisition of task-specific cerebellar forward models (Ramnani, 2014), consistent with the task performance forward model observed during pseudo-random tracking. These models, once established and refined by over-training, could be conserved over long period of time without reconsolidation.

## Implications for Generative Models

Recent attempts at a unifying framework of brain function hypothesize that the CNS acts as a predictive machine (Friston, 2010; Picard and Friston, 2014). The brain improves its belief and hypotheses about the world by continuously generating predictions about inputs, comparing those predictions with results and acting to minimize prediction errors. The theory posits that the brain is organized hierarchically into generative models in which higher levels provide predictions to lower level models and the higher levels use sensory prediction errors from the lower level as inputs to update the predictions (Picard and Friston, 2014). In this framework, perception is understood as inferring causes to sensations by minimizing sensory prediction errors, and action is understood as minimizing sensory prediction errors between expected consequences of action and sensations (Friston, 2010; Aggelopoulos, 2015; Barrett and Simmons, 2015; O’Callaghan et al., 2017). The prediction hypothesis and generative model architecture are being applied to a multitude of brain functions including representation of self (Moutoussis et al., 2014; Picard and Friston, 2014), theory of the mind (Picard and Friston, 2014), and mental disorders (Sterzer et al., 2018a,b).

Cerebellar forward internal models have been proposed as an example of generative models (Pickering and Clark, 2014). The connectivity between the cerebellum and cerebral cortex noted above provides the substrate for recursive network interactions between the two structures, and suggest possible candidates for such hierarchical levels. One of the open issues in this framework is the integration of the cerebellar forward models in the larger cognitive architecture, hinging on whether and how cerebellar models integrate context dependent outputs. The observation that the cerebellar cortex encodes simultaneously forward models of arm kinematics and task performance errors supports the hypothesis that the behavioral context is reflected in the cerebellar activity (see Figure 1F). The independence of the kinematic and task specific models suggests that the cerebellum can engage and update combinations of different forward internal models depending on the behavioral context. This could provide a “complete” control mechanism, integrating execution accuracy and outcome, allowing fast execution of complex behaviors in variable contexts.

## Author Contributions

LP and TE jointly wrote the review.

## Conflict of Interest Statement

The authors declare that the research was conducted in the absence of any commercial or financial relationships that could be construed as a potential conflict of interest. The reviewer EL declared a past co-authorship with the authors to the handling editor.
